# Contraceptive care for patients with bigger bodies in the US: Evaluation of a provider training intervention

**DOI:** 10.1016/j.pmedr.2026.103389

**Published:** 2026-01-19

**Authors:** Yasaman Zia, Alejandra Alvarez, Christina Pineda, Connie Folse, Jen Robinson, Alison Edelman, Suzan Goodman, Cynthia C. Harper

**Affiliations:** a Bixby Center for Global Reproductive Health, University of California San Francisco, United States; b Department of Obstetrics, Gynecology, and Reproductive Sciences, University of California San Francisco, United States; c Department of Nursing, University of California San Francisco, United States; d Consultant, University of California San Francisco, United States; e Obstetrics and Gynecology, School of Medicine, Ohio State University, United States

**Keywords:** Contraception, Emergency contraception, Obesity, Overweight

## Abstract

**Objective:**

Weight stigma is widespread in healthcare. Few studies have assessed contraceptive counseling approaches for patients with bigger bodies. We aimed to assess the impact of a training program on changes in counseling knowledge for those delivering care in the US to patients with bigger bodies.

**Methods:**

From January 2024 to January 2025, we surveyed attendees of Continuing Medical Education (CME)-accredited training on delivering contraceptive care to patients with bigger bodies to assess clinical knowledge change.

**Results:**

Most providers lacked knowledge on contraceptive options and emergency contraception dosing and options for patients with bigger bodies at baseline. We found improved knowledge of clinical recommendations for contraceptive care for this population, improved emergency contraception counseling, and enhanced sensitivity in clinical practices both immediately-post and at 3-month post-training.

**Conclusions:**

Patients with bigger bodies deserve accurate clinical information and comprehensive access to contraception. To provide person-centered care for patients with bigger bodies, this training increased knowledge of how to deliver contraceptive care for this population.

## 1 Introduction

Weight stigma is widespread in healthcare; it contributes to lower quality of care and limits access to care for patients with bigger bodies (Alimoradi et al., 2020). Evidence shows providers overemphasize body mass index (BMI), generalize potential risks associated with higher weight, and give little focus to non-weight related health concerns (Ryan et al., 2023). Providers and patients commonly hold misconceptions about a lack of contraceptive safety and presumed ineligibility for people with bigger bodies, including for emergency contraception (EC), oral contraceptive pills, injectables, and intrauterine devices (Jatlaoui et al., 2002; Boyce and Neiterman, 2021). People with bigger bodies may have unmet need for contraceptive care, with many patients reporting inadequate or no contraceptive counseling and some data demonstrating higher rates of unintended pregnancy (Boyce and Neiterman, 2021; McKeating et al., 2015). Few studies have assessed contraceptive counseling approaches for patients with bigger bodies. We aimed to assess the impact of a training program on changes in counseling knowledge in selected topics in delivering care to patients with bigger bodies.

## 2 Methods

### 2.1 Study design and population

From January 2024 to January 2025, we surveyed training attendees of a Continuing Medical Education (CME)-accredited training on delivery of contraceptive care for patients with bigger bodies provided through University of California, San Francisco (UCSF, 2025). Five live virtual and in-person trainings took place across the US, with participants from multiple states; most notably Kansas, Missouri, New Mexico, Oklahoma, and Texas. Trainings were intended for the full care team, including providers and clinic staff, and included activities such as self-reflection and group discussions. The training objectives included examining the effects of weight stigma on patients, discussing evidence-based guidelines on the provision of contraception for patients with bigger bodies, and evaluating how various clinic policies and practices can create inclusive spaces for people of all body sizes. Training content covered hormonal and non-hormonal forms of contraception as well as emergency contraception. Sessions walked participants through a composite case study and detailed aspects of a hypothetical clinic experience for someone with a bigger body. The case study highlighted issues surrounding inadequate medical equipment, clinic staff bias, and directive and overly restrictive contraceptive counseling from a provider. Participants discussed instances of weight stigma evidence in the case and explored existing guidance, resources, and potential solutions for providing medically-accurate and evidence-based weight-inclusive care. Emails with a survey link and QR codes were shared both pre- and immediately-post training, with a brief series of items to gauge training impact.

The analysis population is limited to attendees who directly provide contraceptive care or education (self-reported via eligibility survey) and completed both surveys. Among eligible training attendees (*n* = 757), 71% (*N* = 539) completed the pre-survey, including registered nurses and other clinic staff (*n* = 253), advanced practice clinicians (*n* = 212), and physicians (*n* = 74). A total of 424 of the survey sample completed the post-survey (79% of respondents and 56% of all eligible trainees). Nurses and clinic staff completed the post-survey at a relatively higher proportion than did physicians and advance practice clinicians (see Table 1).

**Table 1:** Sample characteristics of US providers and clinic staff attending trainings on contraceptive care for patients with bigger bodies (2024–2025).

	Completed Pre-Survey		Completed Immediate Post-Survey		Completed 3-month Post-Survey	
	N	%	N	%	N	%
**Provider type**						
Physician	74	13.7%	47	11.1%	8	10.4%
Advanced Practice Clinician	212	39.3%	142	33.5%	35	45.5%
Nurse/Medical Assistant/Other	253	46.9%	235	55.4%	34	44.2%
Total	539		424		77	

**Practice Setting**						
Primary Care/Public Health Department	201	37.3%	165	38.9%	17	22.1%
Family Planning Clinic	94	17.4%	54	12.7%	27	35.1%
School-based Health Center	168	31.2%	117	27.6%	16	20.8%
Hospital/Abortion Clinic/Other	76	14.1%	88	20.8%	17	22.1%
Total	539		424		77	

### 2.2 Measures and statistical analysis

We analyzed changes in a series of clinical knowledge variables to estimate the training impact, from BMI and weight practices, to the contraceptive patch, and practices around emergency contraception (see Table 2). We used Poisson regression analysis with generalized estimating equations to generate incidence rate ratios with a log-link function and an independent correlation structure to account for clustering by training. We used a repeated cross-sections approach, including data from all learners completing a pre- or post-survey (Vittinghoff et al., 2012). In adjusted regression models, we controlled for provider type (physician, advance practice clinician, nurse or medical assistant) and practice setting (primary care or public health department; family planning clinic; school-based health center; hospital, abortion clinic, or other). In a subset of participants with surveys completed at 3-month post-training (*n* = 77), we estimated the sustained effect of the training on clinical knowledge. Providers who completed the immediate- and the 3-month post surveys, respectively, were entered into quarterly raffles to win a $250 electronic gift card. We used Stata Version 16 for all analyses.

**Table 2:** Contraceptive knowledge change among US providers and staff attending trainings on delivering care to patients with bigger bodies (2024–2025).

Series of models	n (%) correct response			Regression Results Immediate Post		Regression Results 3-month Post	
	Pre-Survey	Immediate Post-Survey	3-month Post-Survey	IRR (95% CI)	aIRR^a^(95% CI)	IRR (95% CI)	aIRR^a^(95% CI)
Model 1: Body Mass Index (BMI) is an accurate measure of health (Correct answer: Disagree) b	75.5% (406/538)	92.7% (392/423)	87.0% (67/77)	1.23 (1.19–1.27)	1.23 (1.19–1.27)	1.15 (1.05–1.27)	1.12 (1.05–1.20)
Model 2: Weight should be proactively discussed during most visits (Correct answer: Disagree) ^b^	46.7% (251/537)	78.5% (332/423)	76.6% (59/77)	1.68 (1.52–1.86)	1.69 (1.54–1.85)	1.64 (1.44–1.86)	1.57 (1.46–1.68)
Model 3: Contraceptive patch should not be prescribed to a patient with BMI > 30 (Correct answer: Disagree) ^b^	29.6% (157/531)	88.0% (373/424)	86.5% (64/74)	2.98 (2.72–3.25)	3.03 (2.70–3.40)	2.93 (2.57–3.33)	2.83 (2.41–3.32)
Model 4: Is doubling dose of levonorgestrel emergency contraceptive pills an effective strategy for patients with BMI ≥ 35? (Correct answer: No)	31.1% (166/533)	86.5% (358/414)	78.4% (58/74)	2.76 (2.46–3.11)	2.82 (2.56–3.11)	2.52 (1.99–3.18)	2.57 (2.05 – 3.22)
Model 5: Which emergency contraception (EC) methods would you consider for a patient with BMI = 35? (Correct answer: Yes)							
5a. Ulipristal acetate (ella)	58.7% (316/538)	78.8% (334/424)	81.2% (63/77)	1.34 (1.27–1.42)	1.39 (1.33–1.45)	1.39 (1.22–1.59)	1.38 (1.17–1.62)
5b. Copper T380 intrauterine device (IUD) for EC	66.4% (357/538)	82.8% (351/424)	84.4% (65/77)	1.25 (1.16–1.34)	1.29 (1.22–1.35)	1.27 (1.20–1.35)	1.22 (1.06–1.40)
5c. Levonorgestrel 52 mg IUD for EC	36.3% (195/537)	64.8% (274/423)	55.8% (48/77)	1.78 (1.62–1.96)	1.91 (1.73–2.10)	1.72 (1.64–1.79)	1.54 (1.30–1.85)

Abbreviations: BMI: body mass index; EC: emergency contraception; IRR: incidence rate ratio; IUD: intrauterine device.

a Adjusted for provider type and practice setting.

b Categorization of responses: Disagree/Strongly disagree vs. Agree/Strongly agree/Don't know.

The Institutional Review Board at University of California, San Francisco approved the study (#12–10,336) and all participants provided informed consent.

## 3 Results

Results showed increased awareness among providers and clinic staff of care for patients with bigger bodies, including improved knowledge that Body Mass Index (BMI) can be an inaccurate measure of health (75.5% to 92.7%, adjusted incidence rate ratio (aIRR): 1.23, 95% confidence interval (CI): 1.19, 1.27) and that weight should not be proactively discussed in every clinic visit (46.7% to 78.5%, aIRR: 1.69, 95% CI: 1.54, 1.85). Providers and clinic staff exhibited increased clinical knowledge of the evidence for counseling patients with bigger bodies in several areas, including that contraceptive patch may be prescribed to a patient with BMI >30 (29.6% to 88.0%, aIRR: 3.03, 95% CI: 2.70, 3.40) (Edelman and Kaneshiro, 2025; Nelson et al., 2021) and that doubling the dose of the levonorgestrel emergency contraceptive pill does not improve efficacy for patients with BMI ≥35 (31.1% to 86.5%, aIRR: 2.82, 95% CI: 2.56, 3.11) (Edelman et al., 2022). Lastly, results for clinical practice questions highlighted important increases in offering a range of emergency contraceptive options to patients with bigger bodies, including the ulipristal acetate pill (58.7% to 78.8%, aIRR: 1.39, 95% CI: 1.33, 1.45), the copper intrauterine device (IUD) as emergency contraception (66.4% to 82.8%, aIRR: 1.29, 95% CI: 1.22, 1.35), and the levonorgestrel IUD as emergency contraception (36.3% to 64.8%, aIRR: 1.91, 95% CI: 1.73, 2.10; Table 2). In a subset of participants with 3-month data, we found a similar magnitude of clinical knowledge gains sustained over time (Table 2).

## 4 Discussion

Patients with bigger bodies deserve comprehensive access to and accurate clinical information on contraception, including emergency contraception. The baseline data indicated some important gaps that were addressed by this training. At baseline, most providers lacked the knowledge that the contraceptive patch may be prescribed to a patient with a bigger body, which can reduce person-centered options for contraception. The majority also believed at baseline that it was appropriate to double the dose of levonorgestrel EC pills for patients with bigger bodies, which is not evidence-based (Edelman et al., 2022). Additionally, less than half of providers would consider levonorgestrel IUD as emergency contraception for patients with bigger bodies, which restricts this option from patients.

## 5 Conclusion

To provide person-centered care for patients with bigger bodies, this training increased knowledge of clinical recommendations for contraceptive care for this population, improved emergency contraception counseling, and gave indications of enhanced cultural-sensitivity in clinical practices as well as awareness of patient-centered discussions of weight or BMI. Knowledge gains were sustained at 3-month post-training among a subset of attendees, which may be indicative of translating clinical knowledge to clinical practice changes. These results may be an over-estimation of knowledge changes as 3-month response rates had loss to follow-up, with higher non-response among clinicians, who also had higher initial knowledge levels than other provider types, and differential non-response among hospital, abortion, and other grouping as compared to other clinical practice settings. Research and eligibility guidelines continue to evolve clinical practice around contraception for patients with bigger bodies (Nguyen et al., 2024). Professional education and training programs are more effective at reducing weight bias when offered early in and regularly throughout the health professional's career (Talumaa et al., 2022).

## AI statement

Generative AI tools were not utilized at any point in the preparation of this work.

## CRediT authorship contribution statement

**Yasaman Zia:** Writing – original draft, Methodology, Investigation, Formal analysis, Conceptualization. **Alejandra Alvarez:** Writing – review & editing, Formal analysis, Data curation. **Christina Pineda:** Writing – review & editing, Formal analysis. **Connie Folse:** Writing – review & editing, Project administration. **Jen Robinson:** Writing – review & editing, Project administration. **Alison Edelman:** Writing – review & editing, Project administration. **Suzan Goodman:** Writing – review & editing, Supervision, Project administration. **Cynthia C. Harper:** Writing – review & editing, Supervision, Investigation, Funding acquisition, Conceptualization.

## Funding statement

This work was supported by the Freedom Together Foundation (2021–2688) and an Anonymous Donor.

## Declaration of competing interest

The authors declare that they have no known competing financial interests or personal relationships that could have appeared to influence the work reported in this paper.
